# Assigning computed tomography involvement score in COVID-19 patients: prognosis prediction and impact on management

**DOI:** 10.1259/bjro.20200024

**Published:** 2020-08-20

**Authors:** Nilu Malpani Dhoot, Usha Goenka, Somali Ghosh, Surabhi Jajodia, Rashmi Chand, Sanjib Majumdar, Suresh Ramasubban

**Affiliations:** 1 Department of Clinical Imaging and Intervention Radiology, Apollo Gleneagles Hospital, Kolkata, West Bengal, India; 2 Department of Critical Care, Apollo Gleneagles Hospital, Kolkata, West Bengal, India

## Abstract

**Objective:**

Chest CT can provide a simple quantitative assessment of the extent of the parenchymal opacities in COVID-19 patients. In this study, we postulate that CT findings can be used to ascertain the overall disease burden and predict the clinical outcome.

**Methods:**

In this prospective study undertaken from March 28, 2020, until May 20, 2020, 142 patients with CT features suggestive of viral pneumonia, and positive RT-PCR for COVID-19 were enrolled. A dedicated spiral CT scanner was used for all COVID-19 suspects. CT features were reported as typical, indeterminate, or atypical for COVID-19 pneumonia. A CT involvement score (CT-IS) was given to each scan and assigned mild, moderate, or severe category depending on the score range. The patients were followed up for at least 15 days.

**Results:**

Ground glass opacity was present in 100% of the patients. There was a significant association between CT-IS and the final outcome of the patients. A statistically significant increasing trend of mortality and requirement of critical medical attention was observed with the rising value of CT-IS in COVID-19.

**Conclusion:**

The severe CT-IS score group has a high mortality. The CT-IS score could be valuable in predicting clinical outcome and could also be useful in triage of patients needing hospital admission. In situations where healthcare resources are limited, and patient load high, a more careful approach for patients with higher CT-IS scores could be indispensable.

**Advances in knowledge:**

CT-IS is a simple quantitative method for assessing the disease burden of COVID-19 cases. It can be invaluable in places with limited resources and high patient load to segregate patients requiring critical medical attention.

## Introduction

The World Health Organization declared novel Coronavirus disease 2019 (COVID-19) a pandemic and public health emergency of international concern on March 11, 2020.^1^ The disease has infected 5,6503,804 individuals and caused 346,775 deaths worldwide, as of May 25, 2020, spreading to more than 210 countries.^2^ All symptomatic and asymptomatic patients are not tested everywhere; thus, underestimating the overall disease burden. A timely diagnosis of the novel COVID-19 is critical for disease management and containing the spread.

The standard of reference for confirming COVID-19 relies on microbiological tests such as real-time polymerase chain reaction (RT-PCR) or sequencing^3,4^ methods on specimens collected from the respiratory tract. The sensitivity of the RT-PCR test done on throat swab samples taken at the first visit is restricted to about 30 to 70%.^5–8^ This is due to the difficulties in collecting the samples under ideal conditions, transporting them to qualified laboratories for examination, the time taken to obtain a result, as well as the limitations of the test kit itself.

Compared to the RT-PCR, chest CT imaging might be a more reliable, practical, and a rapid method to diagnose and assess COVID-19.^9^ CT can be used as a significant adjunct to RT-PCR for identifying and quantifying COVID-19 pneumonia in the present epidemic context,^6,7^ although most radiology organizations and societies have recommended against performing screening CT for the diagnosis of COVID-19.^10,11^ Moreover, when the viral load is low, RT-PCR can be false negative, whereas CT shows lung parenchymal changes.^6,7^ This case scenario warrants repeat RT-PCR before labeling the patient uninfected. An article based on a colossal series of 1014 patients reported 97% sensitivity of lung CT in diagnosing COVID-19. The interval time between negative initial and positive subsequent RT-PCR was about 5 days.^6^ CT can be a vital tool in the primary detection and management of COVID-19 pneumonia,^12^ for symptomatic patients,^7^ especially when the immediate availability of the RT-PCR kits is limited.

In this study, we aim to postulate that CT chest could be used to provide a qualitative and quantitative assessment of the parenchymal changes in COVID-19 patients and that this could further be used to define the disease burden and predict the clinical outcome.

## Methods and materials

### Patient profile

A prospective study was conducted from March 28, 2020, until May 20, 2020, in our tertiary referral hospital. The patient inclusion criteria were symptomatic patients having positive RT-PCR for COVID-19 and CT findings suggesting viral pneumonia. A total of 148 consecutive patients were enrolled. The exclusion criteria were patients with known lung malignancy, interstitial lung disease and active tuberculosis. Six **such** patients were excluded from our study. The maximum interval between CT Chest and RT-PCR was 48 h. The institutional ethical committee approved our research, and informed consent was taken from the patients.

All patients underwent a prescreening questionnaire about COVID-19 symptoms in the triage clinic located outside our Emergency Department. Patient demographics and specific clinical information like fever, cough, dyspnea, duration of symptoms, travel history, contact with Covid-19 positive patient, and co-morbidities were recorded. Fever was defined as a temperature above 98.6 °F.

Both CT chest and RT-PCR were done in most of the COVID-19 suspects. CT chest was the initial investigation in the majority of our patients because of its ready availability with the feasibility of immediate opinion. CT was performed in a dedicated CT machine for all Covid-19 suspects with all precautionary measures to avoid cross-infection. The nasopharyngeal/oropharyngeal swabs were obtained for the RT- PCR (QIAGEN, Shanghai, China) test to confirm the presence of SARS-COV2 infection. The patient’s chest CT findings were reported as per the Radiological Society of North America Consensus Statement.^13^ In the cases having chest CT with typical or indeterminate^13^ manifestations for COVID-19, if the first RT-PCR was negative, a second throat swab for RT-PCR was performed. These patients were considered negative for COVID-19 infection, after two consecutive negative RT-PCR results, but were still advised home quarantine for 14 days.

COVID-19 patients with mild symptoms (fever <100 F, no dyspnea, and maintaining more than 95% oxygen saturation in room air) were discharged from our hospital and sent to dedicated isolation centers. Patients with severe symptoms (fever >100F, severe dyspnea, not maintaining oxygen saturation above 95% in room air) were hospitalized. Data about hospitalization and home/isolation were also collected.

### CT protocol

All CT examinations were obtained with the dedicated CT scanner for Covid-19 suspects using Aquilion Prime 160 CT system (Toshiba, Tokyo, Japan) helical mode scanner. The tube voltage was at 120 kVp, and tube current was set to a low dose of 50 mAs. Low dose CT was used to reduce the radiation dose to the patients. The images were obtained from the thyroid gland level up to the level of the pancreas. No contrast was administered. The scan was captured in the end-inspiratory phase, whenever it was possible for the patient to hold the breath adequately. The slice thickness was 0.8 mm.

The images were analyzed by two radiologists in consensus, having more than 10 years of experience in chest radiology, in both lung (window width 1500 Hounsfiled unit, HU; level, −700 HU) and mediastinal (window width 350 HU; level, 40 HU) settings. Multiplanar reconstructions in the coronal, sagittal, and oblique planes were also performed and read in addition to the axial sections whenever required. The radiologists were blinded to clinical and laboratory findings.

### Image analysis

The CT images were analyzed qualitatively and quantitatively.

The chest CT was assessed for the presence of ground-glass opacities (GGOs), consolidation, crazy paving pattern, reverse halo sign, vessel engorgement, and architectural distortion with subpleural bands. The other features like nodular infiltrates, tree in bud pattern, and cavitation was also noted. Hilar, mediastinal lymphadenopathy and pleural abnormalities were recorded. The structured reporting pattern suggested by the Radiological Society of North America Consensus Statement was followed.^13^

The involvement of the lung was categorized as unilateral or bilateral. The shape of GGO or consolidation was recorded as rounded or coalescing. The predominant mode of distribution was noted as central (inner two-third of the lung) or peripheral (outer one-third of the lung). The presence of any lobar (upper, middle, or lower) predominance was also noted.

GGO was defined as hazy, ill-defined areas of increased attenuation without obscuration of underlying vessels. When GGO was associated with septal thickening, it was called a crazy-paving pattern. Consolidation was defined as homogenous opacification of the lung parenchyma with obscuration of the underlying vessels with or without air bronchogram. Reversed halo sign was characterized by a central GGO or low attenuation surrounded by denser air space consolidation in the shape of a crescent or a ring. Fibrosis, volume loss, and subpleural fibrotic strands were noted as architectural distortion and subpleural bands. Dilatation of the subsegmental vessels to 3 mm or more was read as vessel engorgement. The tree in bud pattern was identified as multiple areas of centrilobular nodules with a linear branching pattern.^14^

### CT involvement score (CT-IS)

Based on the study model by Chung^15^ each of the five lung lobes was assessed for the degree of air space opacification (GGO/consolidation) by the disease:

Score 1 – < 5% involvement.

Score 2 – 5–25% involvement.

Score 3 – 26–49% involvement.

Score 4 – 50–75% involvement.

Score 5 – >75% involvement.

The total CT-IS was the sum of the individual lobar scores ranging from 0 (no involvement) to 25 (maximum involvement, when all the five lobes showed more than 75% involvement). The overall lung score out of 25 was classified as mild, moderate, and severe, depending on the score range. The score between 0 and 9 was taken as a mild disease, 10–17 was taken as moderate disease, and the score range of 18–25 was taken as severe disease.

### Follow-up

The patients were followed up for at least 15 days. The outcome of each patient was noted whether discharged, expired, or still admitted in the hospital. It was also noted if the patient required oxygen support, ventilation, or advanced medical care during the hospital stay. Telephonic follow-up was taken from the patients who were in the isolation centers or home quarantine. Statistical analysis was performed to see if the CT-IS had predictive value in the clinical outcome of the patient.

### Statistical analysis

Data analysis was carried out with the help of Epi Info TM7.2.2.2. Epi Info is a trademark of centers for Disease Control and Prevention (CDC, Atlanta, GA). Descriptive statistical analysis was performed to calculate the means with corresponding standard deviation (SD). The test of proportion was used to compare the different proportions, and the chi-square test was performed to find the associations. The receiver operating characteristic (ROC) curve was used to find the value of CT-IS in predicting the disease outcome. Death or hospital admission requiring advance life support was taken as critical disease outcome. The value of *p* < 0.05 was considered statistically significant. A time to event analysis for end point survival/recovery was performed using the Kaplan–Meier survival analysis.

## Results

### Demographics and clinical analysis

**Figure 1.**
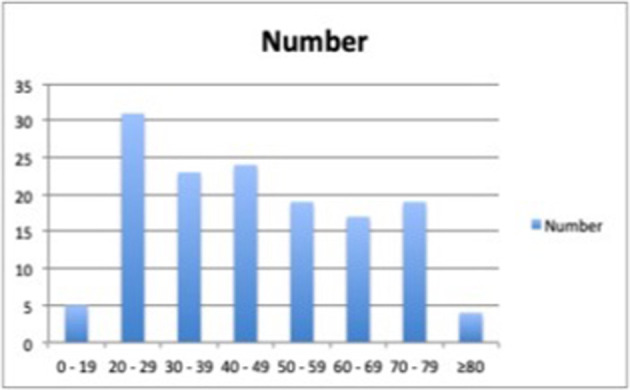
The bar diagram showing age range and number of patients. The x-axis shows the age range in years, and the y-axis indicates the number of COVID-19 patients in that age group.

The bar diagram (Figure 1) shows the number of patients in different age range of COVID-19 cases.

The mean age (Mean ± SD) of the patients was 46 ± 19 years with a range of 15–85 years. Most of the patients (54.9%) were in the age group between 20 and 49 years. The ratio of male and female (Male: Female) was 1.5:1.0, with 59.9% of the patients were males, while 40.1% were females.

Fever (47.2%) followed by cough (30.3%), and breathlessness (29.6%) were most common among the symptoms of Covid-19 infection. Table 1 enumerates all the symptoms. Many patients had more than one symptom. We did not have any patient who had both RT-PCR and chest CT positive and was asymptomatic. The mean duration of symptoms (Mean ± SD) of the patients at presentation was 5.45 ± 4.41 days, with a range of 1–21 days and the median was 4 days.

**Table 1.** Shows the symptoms and number of patients presenting with them

SYMPTOMS	NUMBER OF PATIENTS	PERCENTAGE
Fever	67	47.2%
Cough	43	30.3%
Breathlessness	42	29.6%
Weakness/bodyache	40	28.1%
Sore throat	30	21.1%
Headache	30	21.1%
Loss of smell	10	7%
Diarrhea	3	2.1%

Many patients had co-morbidities. Table 2 shows the type of comorbidity and the number of patients having it. 67 patients (47.1%) had one or more comorbidities. 75 patients (52.8%) had no co-morbidity.

**Table 2.** Shows types of co-morbidities and the number of patients

Co-morbidity	Number of patients	Percentage
Diabetes	46	32.1%
Hypertension	38	26.5%
Malignancy other than lung malignancy	5	3.5%
Chronic renal disease	4	2.8%
Heart disease	3	2.1%
Chronic liver disease	2	1.4%
Immunocompromised	2	1.4%
Inflammatory bowel disease	1	0.7%
Pancytopenia	1	0.7%
Chronic obstructive pulmonary disease	1	0.7%

Four patients (2.8%) had chronic renal disease. Five patients (3.5%) had malignancy other than lung malignancy. 38 (26.5%) patients had hypertension and 46 (32.1%) patients had diabetes. Patients presenting with mild symptoms (52.1%) were more than those with severe presentation (47%). Hospital admission was advised in 63.4% of patients, and 36.6% of the patients were offered isolation. 10 patients died (7%), 49 (34.5%) patients required oxygen, intravenous medications, or life support measures, while 93 (65.5%) required only minor symptomatic treatment.

### CT analysis

GGO ( Table 3) was the most prevalent finding, present in 100% of our cases, followed by vessel engorgement and consolidation. The air space opacities were predominantly rounded (52.8%). The distribution of the parenchymal abnormalities was mainly peripheral (Table 4)and lower lobar. Pleural effusion was noted only in five patients, two of which had chronic renal disease. One patient had mediastinal lymphadenopathy. We did not find centrilobular nodules/tree in bud infiltrates in our cohort. All our reports used the language and classification suggested by the RSNA consensus statement.^13^ While most of our patients had a typical appearance for COVID-19 pneumonia (69, 48.5%), indeterminate findings were present in a lesser number of cases (58, 40.8%), and atypical findings were found in the least number of our patients (15, 10.5%). We did not include patients with negative CT in our study. The ROC curve (Figure 4) shows the value of CT-IS in predicting COVID-19disease outcomes.

**Table 3.** shows the various morphological CT findings in Covid-19 pneumonia.

LUNG FINDING	PATIENTS *N* = 142	PERCENTAGE
GGO(Figures 2 and 3)	142	100%
Crazy paving pattern (Figure 2)	85	59.8%
Consolidation (Figures 2 and 3)	102	71.8%
Reverse halo sign.(Figure 2)	6	4.2%
Vessel engorgement.(Figure 2)	126	88.7%
Architectural distortion/Subpleural bands. (Figure 2)	56	39.4%
Shape-rounded(Figure 2)	75	52.8%
Centrilobular/tree in bud infiltrates	0	0%

GGO, ground glass opacity.

**Figure 2.**
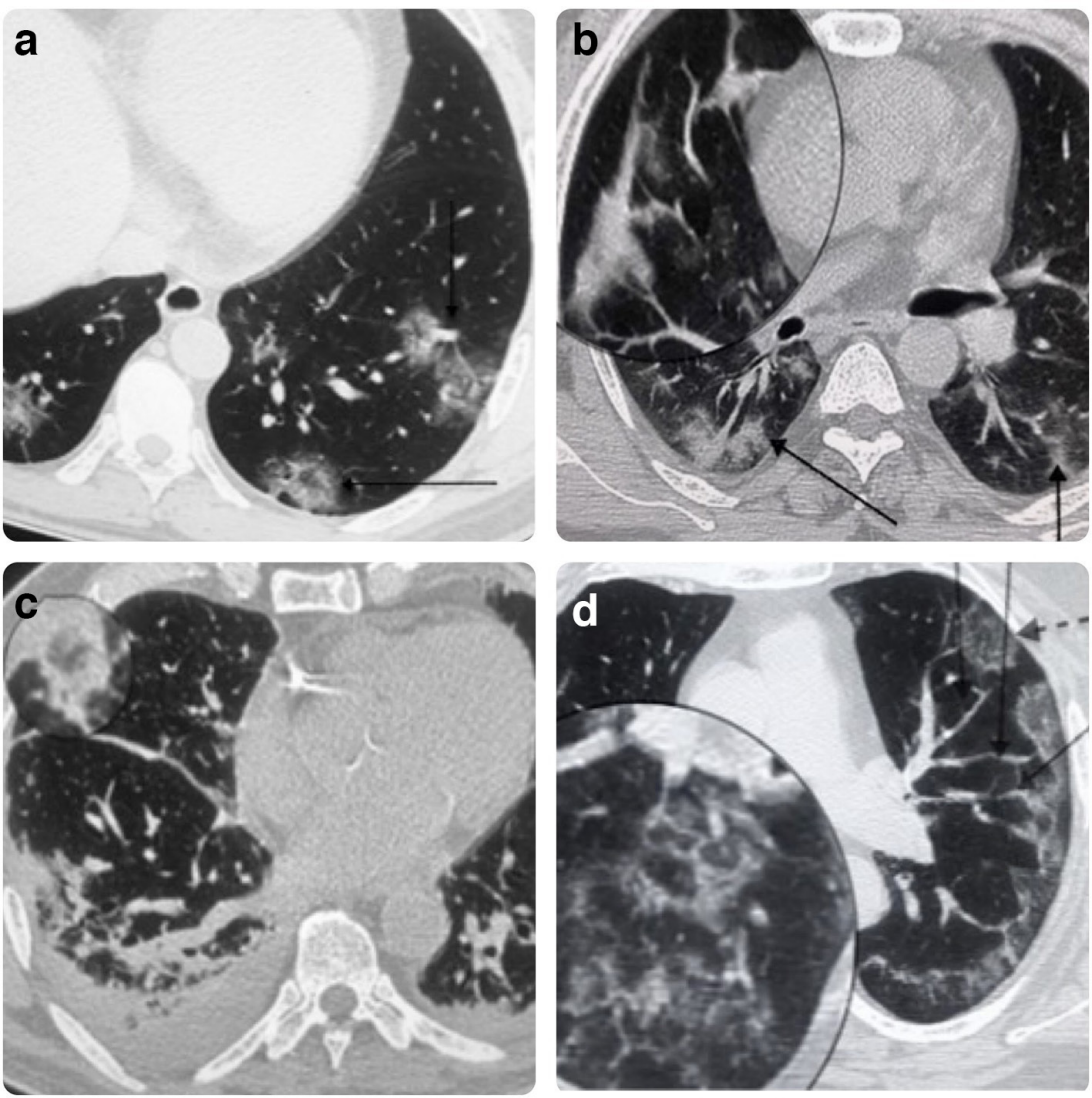
A: Low-dose CT chest shows bilateral rounded GGO in the lower lobes. The vertical arrow points to the vessel engorgement, and the horizontal arrow points to the Reverse Halo sign. B: Chest CT lung window, the magnified section shows the subpleural bands, vertical arrow points to consolidation with GGO, and the inclined arrow points to an area of predominant consolidation. C: Chest CT lung window, the magnified section shows the Reverse halo sign. There is bilateral mild pleural effusion with area of predominant consolidation.D: Axial Section Lung window shows, rounded and patchy peripheral GGO. The interrupted arrow points to rounded shaped GGO. The solid arrows point to vessel engorgement. The magnified section shows the crazy paving pattern. GGO, ground glass opacity.

**Figure 3.**
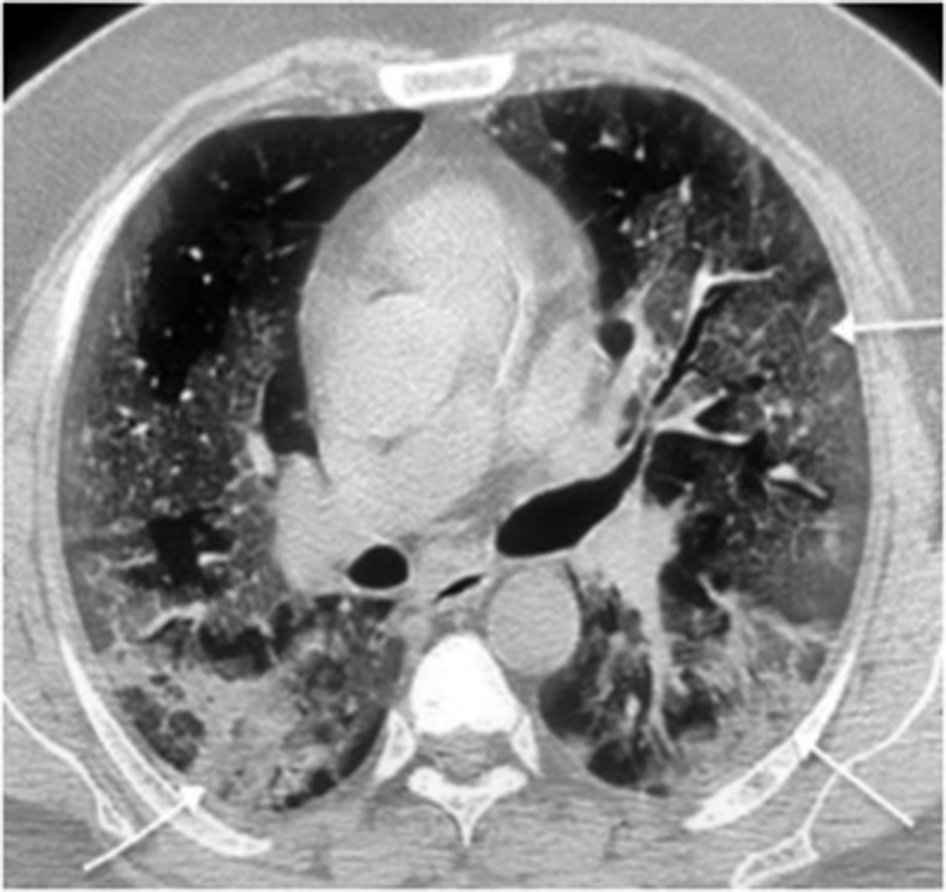
Chest CT, lung window axial section shows coalescing ground glass opacities pointed by the horizontal arrow. The slanting arrows point to bilateral areas of predominant consolidation.

**Table 4.** shows the distribution pattern of lung opacities /pleural and mediastinal abnormalities.

Distribution pattern	Patients *N* = 142	Percentage
Bilateral lung involvement (Figures 2 and 3)	123	86.6%
Peripheral distribution (Figure 2)	126	88.7%
Lower lobe predominance(Figure 2)	129	90.8%
Pleural effusion (Figure 2)	5	3.5%
Mediastinal lymphadenopathy	1	0.7%

CT-IS, CT involvement score; GGO, ground glass opacity; RSNA, Radiological Society of North America.

**Figure 4.**
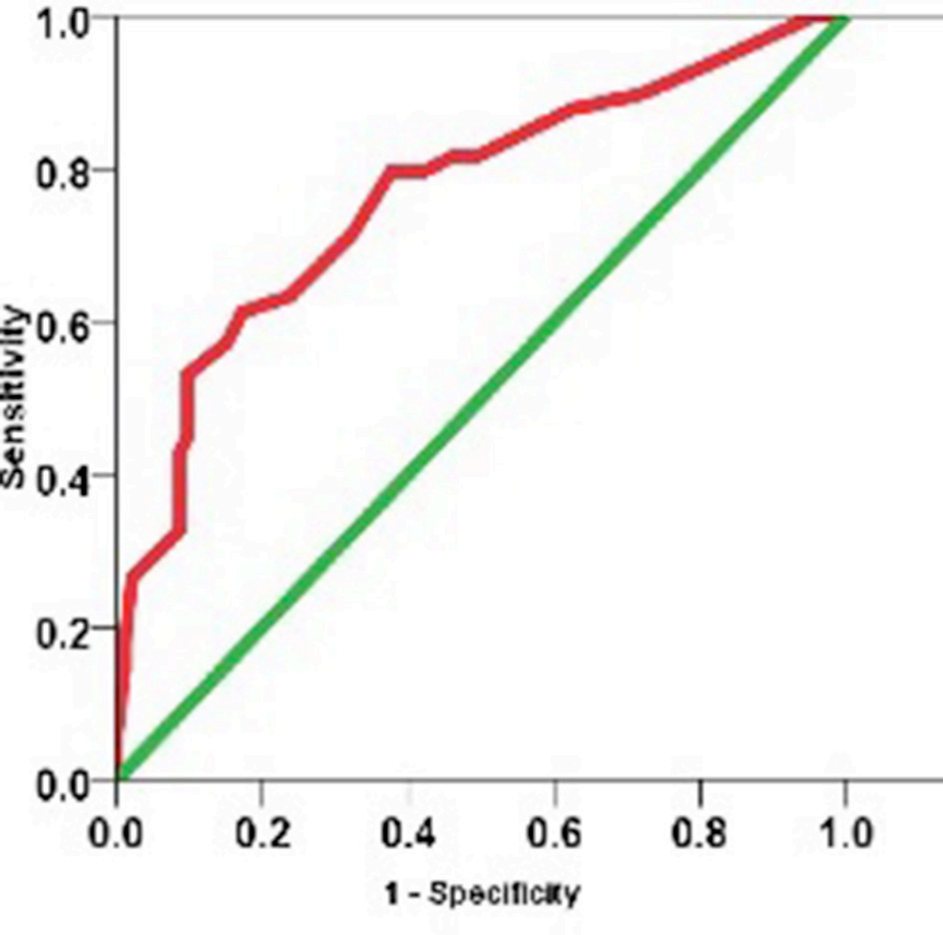
ROC curve of severe disease outcome and CT-IS. CT-IS, CT involvement score; ROC, receiver operating characteristic Table 5

**Table 5.** shows the AUC

AUC	Std. error(a)	Asymptotic sig.^b^	Asymptotic 95% confidence interval	
			Lower bound	Upper bound
0.766	0.044	<0.0001	0.680	0.852
a. Under the non-parametric assumption.				

AUC, area under the curve.

Value of CT-IS for predicting disease severity was found to be 10.28 (*p* < 0.0001)(Figure 4)(Table 5)

Nearly half (65, 45.8%) of our patients had a CT-IS Table 6 below 10, in the mild range {0–9}. The rest of the patients (77, 54.2%) had CT-IS of 10 and above and were further divided into the moderate range {10–17} and severe range {18–25}. The total number of patients in the moderate category was 51 (35.9%), while 26 (18.3%) patients were in the severe category. In the patients having CT-IS in the mild range, 2 (3%, 2/65) patients died, and seven patients (10.7%, 7/65) required critical supportive medical treatment (oxygen support, intravenous medication, or life support). This is significantly (*p* < 0.00001) less than the moderate/severe CT-IS score range.The patients who died in the mild group had underlying co-morbidities: One of them had a treated ovarian malignancy with pancytopenia, and the other had chronic renal disease on maintenance hemodialysis. In the moderate category CT-IS, 1 (1.9%, 1/51) patient died, and 21 (41%, 21/51) patients required critical medical care. The mortality was statistically significant (*p* < 0.001) when compared to severe category. The deceased patient had no co-morbidity. 7 (26.9%, 7/26) patients died in the severe range of the CT-IS, and 11 (42%, 11/26) patients required critical medical attention. Three out of seven patients who died had co-morbidities: two of them had diabetes and hypertension, and one had only diabetes. Overall, CT-IS correlates well with patient clinical outcomes, including mortality. χ^2^ test showed that there was a significant association between higher CT-IS and the final grievous outcome of the patients (*p* < 0.00001). A statistically significant increasing trend of mortality and requirement of critical medical care was observed with the rising value of CT-IS. The median time between CT scan and death of the patient was 10 days.

**Table 6.** Shows the patients with CT-IS in different range with patient demographics and clinical outcome

CT-IS	Mild (0–9)	Moderate (10-17)	Severe(18-25)
Total Patients	65 (65/142, 45.8%)	51 (51/142, 35.9%)	26 (26/142,18.3%)
Age(Mean)	39	34	48
Sex(%)	F (53.8%) M (46.1%)	F (68.6%) M (31.3%)	F (57.6%) M (42.3%)
Total Recovered	63 (96.9%)	50 (98%)	19 (73%)
Recovered with mild medical care	56 (86.15)	29 (56%.8%)	11 (42.3%)
Recovered with critical medical care	7 (10.7%)	21 (41.1%)	8 (30.7%)
Deceased	2 (3%)	1 (1.9%)	7 (26.9%)

CT-IS, CT involvement score.

**Figure 5.**
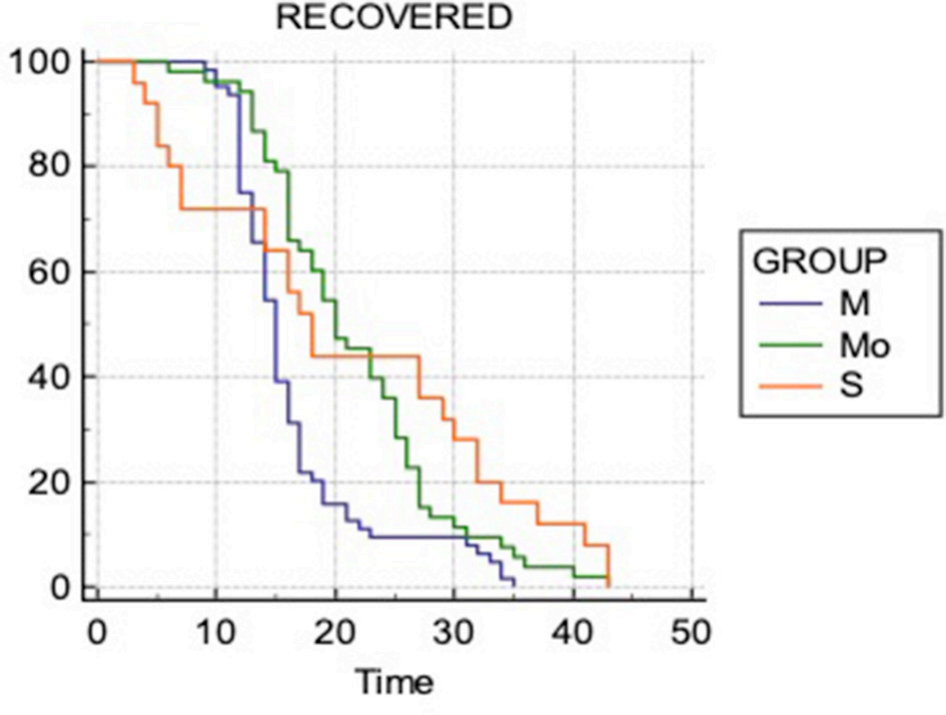
Kaplan–Meier Analysis. M- Mild CT-IS range, Mo-Moderate CT-IS range and S- Severe CT-IS range. The x-axis is time interval in days and the y-axis is cumulative probability of survival/recovery. CT-IS, CT involvement score.

A time-to-event analysis for the end point of recovery/survival was performed using the Kaplan–Meier survival analysis (Figure 5).Log rank test showed that there was significant difference between lengths of time for recovery of the patients with CT-IS score of mild, moderate severe groups (*p* = 0.0002). The patients with mild CT-IS score recovered earlier than patients with higher CT-IS scores of moderate and severe. The median time to recovery for mild range was 15 days while 18 and 20 days for moderate and severe range.

## Discussion

Detection of COVID-19 at an early stage is crucial to prevent the rapid spread of this highly contagious infection and to treat the symptoms. Protocols for disease containment and patient management depend primarily on disease diagnosis.^16–18^ Unfortunately, COVID-19 laboratory testing has been compromised by restricted recourses and inherent limitations of the nucleic acid kits.^19^ Chest Radiographs lack high sensitivity and specificity, leading to a large number of false negatives.^20^ CT chest is more sensitive than chest radiography, showing lung opacities even at an initial stage.^21,22^ In this prospective study, we have studied 142 chest CT's of RT-PCR positive COVID-19 patients. We assessed the images qualitatively and quantitatively and assigned each patient a CT-IS. Follow-up of these patients revealed that the high CT-IS positively correlated with increased disease severity and mortality.

Old age and co-morbidities make an individual more prone to contracting the disease and also to increased disease severity.^23^ Patients with COVID-19 frequently develop symptoms of fever (98%) and cough (76%).^4^ Similar to the literature, in our study cohort, the elderly population and patients with co-morbidities had high mortality, and also the most prevalent presenting symptom was fever followed by cough.

Multiple recent pieces of literature have described the characteristic CT features of COVID-19. This includes GGOs (57–100%), bilateral involvement (76–88%), and peripheral distribution (33–85%).^15,24–27^ Consolidation and crazy paving pattern are also published common findings.^21,23,28^ The majority of patients show involvement of multiple lobes, particularly the lower lobes in COVID-19 pneumonia.^29^ The results of our study are also consistent with the above literature. We found GGOs in 100% of our patients, which was the hallmark finding in our patients. Consolidation was present in about 72% of our cases. The distribution was predominantly peripheral (89%) and lower lobar (91%).

A fascinating CT manifestation was the presence of enlarged subsegmental pulmonary vessels in 88.7% of our patients. This finding was previously described by Albarello et al and Damiano et al..^27,30^ Bai et al also described vascular enlargement in 59% of their patients with COVID-19 pneumonia and 22% of those with non-viral non-COVID-19 pneumonia..^31^ Ye et al suggested that vascular enlargement might be due to pro-inflammatory factors.^32^ Architectural distortion, subpleural bands, and reverse halo sign were also noticed in COVID-19 infection by other authors^33^ in concurrence with our study. We qualitatively analyzed our CT, reported them as typical, indeterminate, or atypical based on Radiological Society of North America consensus guidelines,^13^ thus decreasing reporting variability and uncertainty in reporting findings. This format also conveyed to our clinicians our confidence in suggesting COVID-19 pneumonia. The RT-PCR test was repeated within 48 h if the first throat swab for COVID-19 was negative in patients having chest CT reported as Typical or Indeterminate.

CT findings are indistinguishable from those of viral pneumonia in general^25,34,35^ , lacking the specificity for COVID-19. It is therefore arduous to distinguish COVID-19 from other viral infections on imaging. Even though CT abnormalities in COVID-19 pneumonia more regularly manifest a peripheral predominance, with rarely pleural effusion and lymphadenopathy^31^ CT features overlap largely. However, a chest CT revealing features of viral pneumonia, it is only wise to take it as COVID-19 infection in the current pandemic unless proved otherwise. In our patients having atypical CT findings, five patients (3.5%, 5/142) had pleural effusion, and one patient had mediastinal lymphadenopathy. While pneumothorax was reported in one patient with confirmed COVID-19 infection in a study^36^ a direct complication of the disease could not be established. None of our patients had a pneumothorax.

We also did a quantitative analysis of the CT findings. We followed a scoring method based on the amount of air space opacification involving five lung lobes, as a surrogate for COVID-19 burden. Our CT-IS was inspired by a study model of the Chung et al.^15^ We further classified the CT-IS range into mild, moderate, and severe groups. We observed that the severe CT-IS group patients had an unfavorable clinical outcome. These patients had increased mortality and frequently required life support. Similarly, the moderate CT-IS group had a worse prognosis compared to the mild CT-IS group. This relatively simple objective method could identify patients with worse prognosis, thereby predicting advanced medical attention, particularly in scenarios of limited availability of healthcare resources. A study has suggested chest CT scores correlate well with the risk factors for mortality over periods, thus they may be used as a prognostic indicator in COVID-19.^37^ A visual or software quantification the extent of CT lung abnormality were predictors of ICU admission or death in another study.^38^

Our study had a few limitations. First, we assessed only the first chest CT obtained at presentation; therefore, the study was not controlled by the number of days since the start of symptoms, which could affect the CT-IS. Second, the CT-IS was investigated only in symptomatic patients with positive CT and RT-PCR and in a single tertiary care center; our small group of patients had more co-morbidities than the general population; hence our study showed a higher mortality rate. Third, due to the urgency of the situation, a few patients' final clinical outcomes were not available at the time of this communication. Finally, it is not possible to exclude the possibility of superinfection in some of the patients. Further research with larger cohorts in multiple centers is still necessary to determine the effectiveness of CT-IS and the proposed prediction of clinical results.

In conclusion, this study provides a simple quantitative method for assessing the disease burden of COVID-19 in the initial chest CT. Patients with a severe CT-IS score have high mortality compared to a patient with a mild or moderate CT-IS score. The CT-IS could be valuable in predicting clinical outcome and could also be useful in triage of patients needing hospital admission and advanced medical attention. In situations where healthcare resources are limited, and patient load high, a more vigilant approach and greater concentration of health resources for patients with higher CT-IS score could be logical and indispensable.
